# Supplementary material for: Independent Validation of a Deep Learning nnU-Net Tool for Neuroblastoma Detection and Segmentation in MR Images
Source: Cancers (Basel). 2023 Mar 6;15(5):1622. doi: 10.3390/cancers15051622 (PMC10000775; doi:10.3390/cancers15051622)
Supplement: Supplementary file 1 [file cancers-15-01622-s001.zip › cancers-2203203-supplementary-Table S1.pdf]

Dataset description		Training-tuning set. n (%)	Independent validation set. n (%)
MRI series	Number	132	535
Age at diagnosis	Mean± SD (months)	37±39	18±32
Gender	Male	62 (47)	145 (48)
	Female	70 (53)	155 (52)
Histology	Neuroblastoma	104 (79)	263 (88)
	Ganglioneuroblastoma	18 (13)	27 (9)
	Ganglioneuroma	10 (8)	10 (3)
Timepoint	At diagnosis	132 (100)	486 (91)
	After treatment	0 (0)	49 (9)
Location	Abdominopelvic	105 (80)	430 (80)
	Cervicothoracic	27 (20)	105 (20)
Manufacturer	GE	51 (39)	105 (20)
	Siemens	54 (41)	318 (59)
	Philips	27 (20)	109 (20)
	Canon	0 (0)	3 (1)
Magnetic field strenght	1.5T	116 (88)	435 (81)
	3T	16 (12)	100 (19)
Sequences	T2wSE	84 (64)	307 (57)
	T2wSE-FS	48 (36)	176 (33)
	T2wSE STIR	0 (0)	41 (8)
	T2*wGE FS	0 (0)	11 (2)
