# Supplementary material for: Independent Validation of a Deep Learning nnU-Net Tool for Neuroblastoma Detection and Segmentation in MR Images
Source: Cancers (Basel). 2023 Mar 6;15(5):1622. doi: 10.3390/cancers15051622 (PMC10000775; doi:10.3390/cancers15051622)
Supplement: Supplementary file 1 [file cancers-15-01622-s001.zip › cancers-2203203-supplementary-Table S2.pdf]

Timepoint	Location	Magnetic field	Weight	Mean	SD	N
Diagnosis	Abdominopelvic	1.5	T2 SE FS	0.837	0.337	101
			T2 GE FS	0.916	0.216	7
			STIR	0.917	0.232	26
			T2 SE	0.893	0.261	185
			All sequences	0.878	0.285	319
		3	T2 SE FS	0.904	0.280	24
			STIR	0.878	0.173	6
			T2 SE	0.929	0.143	37
			All sequences	0.916	0.203	67
		All magnetic fields	T2 SE FS	0.850	0.327	125
			T2 GE FS	0.916	0.216	7
			STIR	0.910	0.220	32
			T2 SE	0.899	0.246	222
			All sequences	0.885	0.272	386
	Cervicothoracic	1.5	T2 SE FS	0.997	0.009	15
			T2 GE FS	0.951	0.093	4
			STIR	0.976	0.032	4
			T2 SE	0.949	0.135	58
			All sequences	0.959	0.117	81
		3	T2 SE FS	0.990	0.019	8
			STIR	1.000	0.000	2
			T2 SE	0.964	0.076	9
			All sequences	0.979	0.054	19
		All magnetic fields	T2 SE FS	0.995	0.014	23
			T2 GE FS	0.951	0.093	4
			STIR	0.984	0.028	6
			T2 SE	0.951	0.128	67
			All sequences	0.963	0.108	100
	All locations	1.5	T2 SE FS	0.858	0.319	116
			T2 GE FS	0.929	0.176	11
			STIR	0.925	0.216	30
			T2 SE	0.907	0.238	243
			All sequences	0.895	0.261	400
		3	T2 SE FS	0.926	0.245	32
			STIR	0.908	0.156	8
			T2 SE	0.936	0.133	46
			All sequences	0.930	0.182	86
		All magnetic fields	T2 SE FS	0.873	0.305	148
			T2 GE FS	0.929	0.176	11
			STIR	0.922	0.203	38
			T2 SE	0.911	0.225	289
			All sequences	0.901	0.250	486
Treatment	Abdominopelvic	1.5	T2 SE FS	0.552	0.438	14
			STIR	0.776	0.295	3
			T2 SE	0.774	0.286	12
			All sequences	0.667	0.374	29
		3	T2 SE FS	0.915	0.083	11
			T2 SE	0.726	0.485	4
			All sequences	0.865	0.251	15
		All magnetic fields	T2 SE FS	0.712	0.375	25
			STIR	0.776	0.295	3
			T2 SE	0.762	0.328	16
			All sequences	0.735	0.347	44
	Cervicothoracic	1.5	T2 SE FS	0.849	0.131	3
			T2 SE	0.979	0.030	2
			All sequences	0.901	0.118	5
		All magnetic fields	T2 SE FS	0.849	0.131	3
			T2 SE	0.979	0.030	2
			All sequences	0.901	0.118	5
	All locations	1.5	T2 SE FS	0.605	0.414	17
			STIR	0.776	0.295	3

			T2 SE	0.803	0.273	14
			All sequences	0.702	0.357	34
		3	T2 SE FS	0.915	0.083	11
			T2 SE	0.726	0.485	4
			All sequences	0.865	0.251	15
		All magnetic fields	T2 SE FS	0.727	0.358	28
			STIR	0.776	0.295	3
			T2 SE	0.786	0.316	18
			All sequences	0.752	0.334	49
All timepoints	Abdominopelvic	1.5	T2 SE FS	0.803	0.361	115
			T2 GE FS	0.916	0.216	7
			STIR	0.903	0.237	29
			T2 SE	0.886	0.263	197
			All sequences	0.861	0.298	348
		3	T2 SE FS	0.908	0.235	35
			STIR	0.878	0.173	6
			T2 SE	0.909	0.200	41
			All sequences	0.906	0.212	82
		All magnetic fields	T2 SE FS	0.827	0.338	150
			T2 GE FS	0.916	0.216	7
			STIR	0.898	0.225	35
			T2 SE	0.890	0.253	238
			All sequences	0.869	0.284	430
	Cervicothoracic	1.5	T2 SE FS	0.973	0.073	18
			T2 GE FS	0.951	0.093	4
			STIR	0.976	0.032	4
			T2 SE	0.950	0.132	60
			All sequences	0.956	0.117	86
		3	T2 SE FS	0.990	0.019	8
			STIR	1.000	0.000	2
			T2 SE	0.964	0.076	9
			All sequences	0.979	0.054	19
		All magnetic fields	T2 SE FS	0.978	0.062	26
			T2 GE FS	0.951	0.093	4
			STIR	0.984	0.028	6
			T2 SE	0.951	0.126	69
			All sequences	0.960	0.109	105
	All locations	1.5	T2 SE FS	0.826	0.341	133
			T2 GE FS	0.929	0.176	11
			STIR	0.912	0.223	33
			T2 SE	0.901	0.241	257
			All sequences	0.879	0.275	434
		3	T2 SE FS	0.923	0.214	43
			STIR	0.908	0.156	8
			T2 SE	0.919	0.184	50
			All sequences	0.920	0.194	101
		All magnetic fields	T2 SE FS	0.849	0.317	176
			T2 GE FS	0.929	0.176	11
			STIR	0.911	0.210	41
			T2 SE	0.904	0.232	307
			All sequences	0.887	0.262	535
