# Supplementary material for: Independent Validation of a Deep Learning nnU-Net Tool for Neuroblastoma Detection and Segmentation in MR Images
Source: Cancers (Basel). 2023 Mar 6;15(5):1622. doi: 10.3390/cancers15051622 (PMC10000775; doi:10.3390/cancers15051622)
Supplement: Supplementary file 1 [file cancers-15-01622-s001.zip › cancers-2203203-supplementary-Table S3.pdf]

Dependent variable: DSC

Variable	F	P value
Timepoint	0.081	0.775
Location	2.561	0.110
Magnetic field	2.374	0.124
Weight	0.107	0.956
Location x Magnetic field	0.171	0.679
Timepoint x Location	1.272	0.260
Location x Weight	0.478	0.698
Timepoint x Magnetic field	3.117	0.078
Magnetic field x Weight	0.794	0.453
Timepoint x Weight	0.383	0.682
